# Radial Artery Pseudoaneurysm With Cutaneous Manifestations in a Patient With Neurofibromatosis Type 1

**DOI:** 10.7759/cureus.76907

**Published:** 2025-01-04

**Authors:** Takahiro Kobayashi, Shin Iinuma, Hitoki Hashiguchi

**Affiliations:** 1 Dermatology, Japanese Red Cross Kitami Hospital, Kitami, JPN; 2 Dermatology, Asahikawa Medical University, Asahikawa, JPN; 3 Cardiovascular Surgery, Hokkaido Prefectural Kitami Hospital, Kitami, JPN

**Keywords:** neurofibromatosis type 1, radial artery pseudoaneurysm, transradial cardiac catheterization, vascular complication, vascular surgery

## Abstract

Neurofibromatosis type 1 (NF1) is an autosomal dominant disorder with a broad spectrum of clinical features, including cutaneous manifestations. Although vascular complications of NF1 are less frequently recognized, they are significant. This report describes a rare case of a radial artery pseudoaneurysm that manifested as a rapidly enlarging erythematous cutaneous nodule in a 43-year-old man with NF1 one year after transradial cardiac catheterization. A clinical examination revealed a pulsatile lesion, and color Doppler ultrasonography and computed tomography angiography confirmed the diagnosis. The delayed presentation of this case underscores the importance of long-term surveillance for patients with NF1, particularly those who have undergone vascular interventions. The identification of atypical skin lesions as potential indicators of underlying vascular pathology can facilitate an early diagnosis and improve the outcomes of patients with NF1.

## Introduction

Neurofibromatosis type 1 (NF1), which is an autosomal dominant hereditary disorder caused by mutations of the NF1 gene, has an incidence of approximately one in 2,500-5,000 individuals. NF1 is clinically heterogeneous and has a wide range of presentations, including café-au-lait macules, axillary and/or inguinal freckling, and neurofibromas [1]. The primary manifestations of NF1 are cutaneous and neural; however, although vascular complications of this condition are significant, they are under-recognized. Vascular abnormalities that occur with NF1, which are collectively termed NF1 vasculopathy, encompass a range of pathologies, including stenosis, occlusion, aneurysms, pseudoaneurysms, and arteriovenous malformations [2]. This report describes a rare case of a radial artery pseudoaneurysm with cutaneous manifestations in a patient with NF1, thus focusing attention on the importance of awareness of vascular complications associated with this condition.

## Case presentation

A 43-year-old man presented with a one-week history of pain and an erythematous skin lesion on the volar aspect of the right wrist. He had a history of café-au-lait macules since birth and multiple neurofibromas that affected his trunk and extremities; therefore, NF1 was diagnosed at age 30 years. At age 43 years, he experienced an acute myocardial infarction caused by a coronary artery aneurysm and underwent cardiac catheterization via the right radial artery. Subsequently, the patient underwent coronary artery bypass grafting and was prescribed maintenance therapy comprising antiplatelet therapy with aspirin. One year after cardiac catheterization, a rapidly enlarging cutaneous nodule developed at the puncture site of the right radial artery. The patient did not have a history of trauma. The lesion was initially diagnosed as a furuncle and treated with oral antibiotics; however, it continued to enlarge.

**Figure 1:**
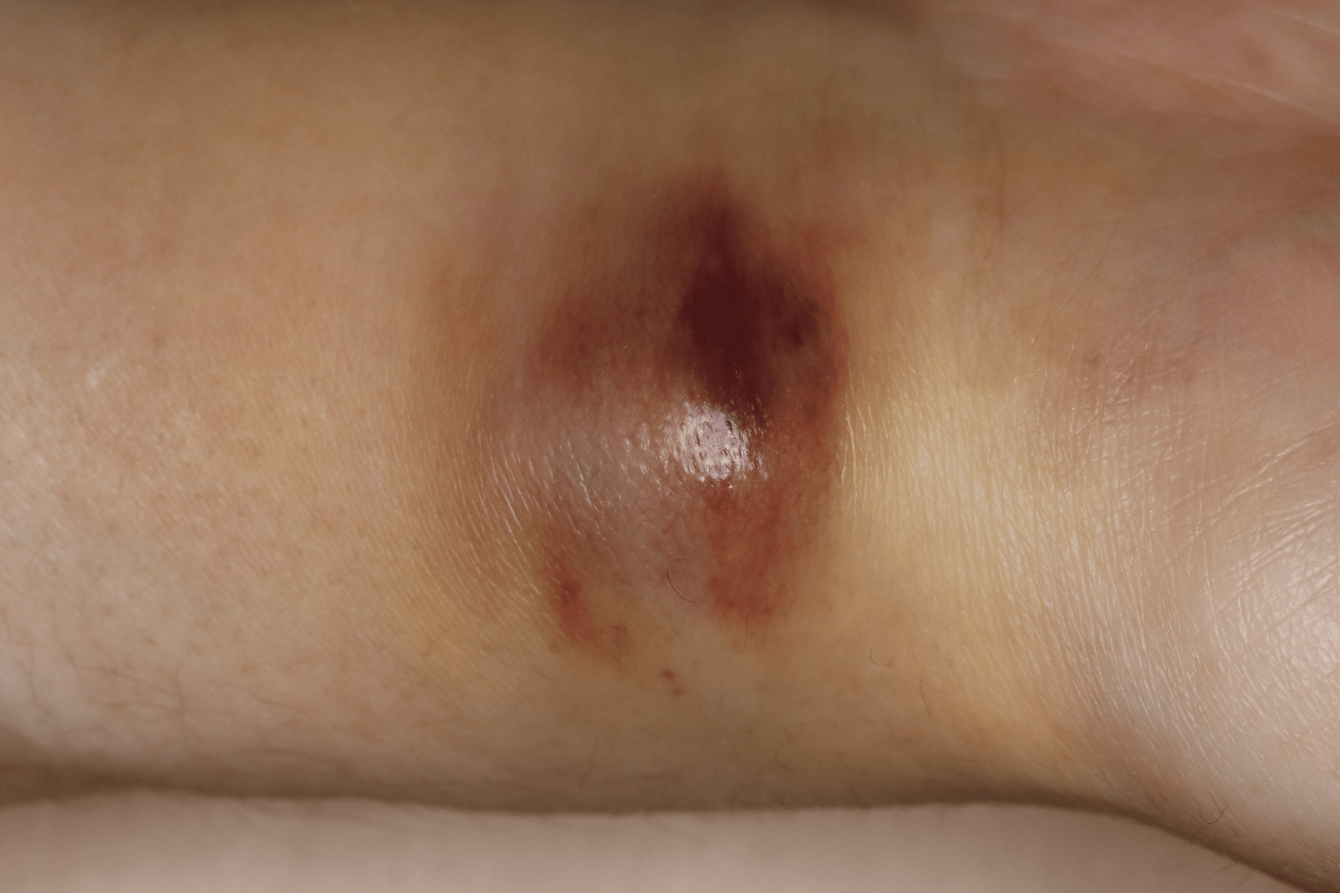
Clinical presentation A 30-mm dome-shaped, erythematous nodule with purpura is observed on the volar aspect of the right wrist.

A physical examination revealed a 30-mm dome-shaped, erythematous nodule with purpura on the volar aspect of the right wrist accompanied by arterial pulsation (Figure 1).

**Figure 2:**
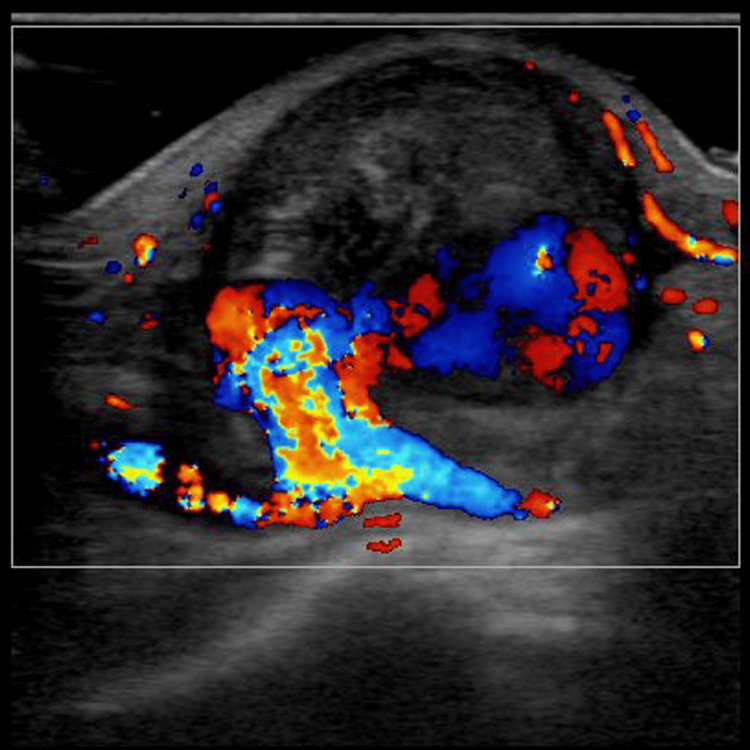
Ultrasonography findings Ultrasonography revealed the presence of a hypoechoic cystic structure with dimensions of 23×20×20 mm in close proximity to the distal radial artery. A color Doppler analysis revealed the presence of blood flow within the lesion that was supplied by the radial artery and exhibited a distinctive swirling motion.

Ultrasonography revealed a hypoechoic cystic structure with dimensions of 23×20×20 mm in close proximity to the distal radial artery. A color Doppler analysis revealed blood flow within the lesion that was supplied by the radial artery and exhibited a distinctive swirling motion (Figure 2).

**Figure 3:**
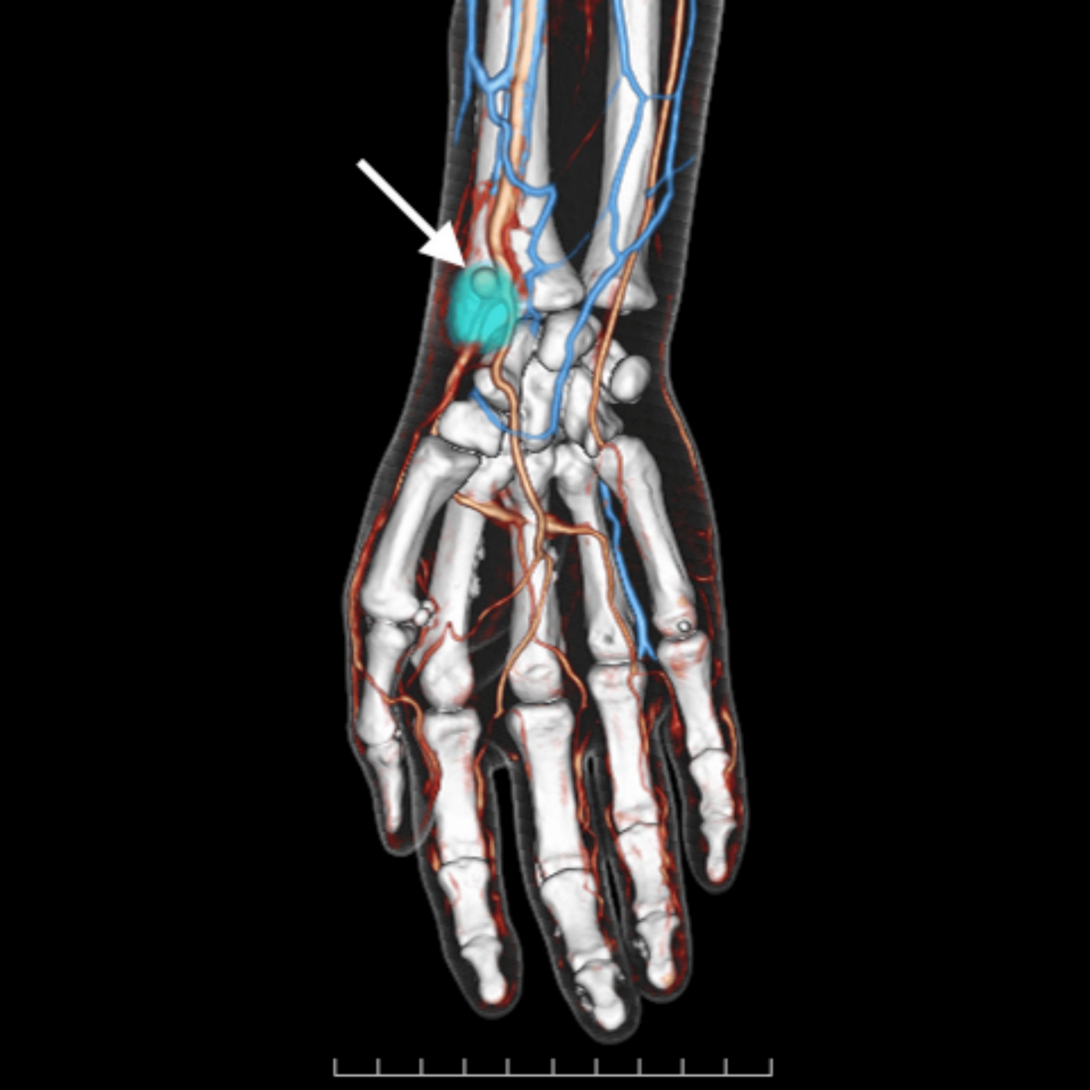
Computed tomography angiography Three-dimensional computed tomography angiography image confirmed the presence of a well-defined contrast collection at the volar aspect of the wrist with direct communication with the radial artery (arrow).

Computed tomography angiography confirmed the presence of a well-defined contrast collection at the volar aspect of the wrist with direct communication with the radial artery, which was consistent with the diagnosis of a pseudoaneurysm (Figure 3).

The patient was subsequently referred for vascular surgery and underwent surgical pseudoaneurysm repair. However, two weeks postoperatively, recurrence of the pseudoaneurysm necessitated radial artery ligation. The patient's postoperative course was uncomplicated.

## Discussion

Vascular complications with NF1 occur in 0.4-6.4% of patients; although specific data regarding pseudoaneurysms are unavailable, this frequency is higher than that of the general population [2]. Although rare, these complications are potentially fatal and typically affect medium to large arteries. Despite their rarity, vascular abnormalities are the second most common cause of death associated with NF1 after malignant transformation of neurofibromas. The aortic, renal, and mesenteric arteries are most commonly affected by these abnormalities. Lesions often develop before age 50 years, and signs such as hypertension should prompt an evaluation to detect NF1-related vasculopathy. However, vascular involvement of the upper extremities is extremely rare, and only a few cases of radial artery aneurysms have been reported [3-5].

The underlying pathophysiology of NF1-related vasculopathy is not completely understood. However, the current evidence suggests that large vessels are susceptible to direct infiltration by adjacent tumors such as schwannomas, resulting in intimal proliferation, media thinning, and fragmentation of elastic tissue [6,7]. These structural changes predispose the vessels to stenosis and aneurysm formation. Additionally, neurofibromin, which is the protein encoded by the NF1 gene, plays a critical role in vascular remodeling [8]. In smaller vessels, wall dysplasia, which is characterized by fibrohyaline thickening of the intima and muscularis, is often observed. These abnormalities further compromise vessel integrity and increase the risk of vascular complications [7].

Radial artery pseudoaneurysms are rare, with an incidence of approximately 0.03-0.09% after transradial catheterization [9]. Pseudoaneurysms arise from lacerations of the arterial wall that allow blood to extravasate into surrounding tissues. The resulting sac is encapsulated by fibrous tissue rather than the normal arterial layers. In contrast to true aneurysms, pseudoaneurysms lack all three arterial wall layers and are prone to rapid expansion and rupture. Pseudoaneurysms present clinically as pain, swelling, and, occasionally, pulsatile masses or bruits. Diagnostic imaging, particularly Doppler ultrasonography, is instrumental in the diagnosis of pseudoaneurysms because their characteristic finding is bidirectional flow caused by the swirling of blood, which is referred to as the yin-yang sign [10].

The formation of radial artery pseudoaneurysms is linked to several risk factors, including repeated arterial puncturing, the use of large sheaths to introduce, guide, and protect catheters during procedures (thereby increasing the risk of arterial trauma and contributing to pseudoaneurysm formation, particularly in patients with vascular fragility), abnormalities in the coagulation process, the use of anticoagulant or antiplatelet medications, and inadequate hemostasis [11]. Pseudoaneurysms can occur between one day and two weeks after arterial catheterization [12]. However, in the current case, the pseudoaneurysm developed one year after catheterization, which is far beyond the typical timeframe and strongly suggests a spontaneous origin rather than a direct relationship with the catheterization procedure. This highlights the importance of recognizing NF1-related vasculopathy as a potential cause of delayed vascular complications. Therefore, long-term monitoring of patients with vascular fragility, such as those with NF1, is necessary.

The objectives of radial artery pseudoaneurysm management are the elimination of arterial communication with the hematoma and the treatment of arterial defects. Noninvasive methods such as ultrasound-guided compression and thrombin injection may be effective for small asymptomatic pseudoaneurysms. However, large or symptomatic pseudoaneurysms, particularly those that do not respond to conservative treatment, often require surgical intervention to prevent rupture and thromboembolic complications. In the present case, progressive sac enlargement and persistent arterial communication resulted in the need for surgical repair [13].

Clinicians should be aware that patients who present with café-au-lait macules and cutaneous neurofibromas, even in the absence of a formal diagnosis of NF1, may be at risk for vascular complications. The presence of unexplained swelling, hemorrhage, or pulsatile masses in such individuals should prompt the consideration of NF1-related vasculopathy, including aneurysmal changes and pseudoaneurysm formation. Early detection and appropriate management of these conditions are essential to mitigating the risk of rupture and subsequent complications. Because of the significant vascular risks of patients with NF1, regular clinical assessments and imaging, including Doppler ultrasonography and computed tomography angiography, are recommended for early detection and monitoring tailored to the individual's risk profile.

## Conclusions

A radial artery pseudoaneurysm is a rare but significant vascular complication that occurs in patients with NF1. The delayed presentation of a pseudoaneurysm in our patient one year after transradial catheterization underscores the vascular fragility of this condition. Cutaneous manifestations are important indicators of systemic complications in patients with NF1. Our patient's initial dermatological consultation for a rapidly enlarging erythematous nodule ultimately led to the identification of a pseudoaneurysm, thus emphasizing the significance of recognizing atypical skin findings as potential markers of underlying vascular pathology. Because of the considerable risks of morbidity and mortality associated with these vascular lesions, long-term monitoring is necessary to improve the outcomes of patients with NF1.
